# The corticolimbic structural covariance network as an early predictive biosignature for cognitive impairment in Parkinson's disease

**DOI:** 10.1038/s41598-020-79403-x

**Published:** 2021-01-13

**Authors:** Yueh-Sheng Chen, Hsiu-Ling Chen, Cheng-Hsien Lu, Chih-Ying Lee, Kun-Hsien Chou, Meng-Hsiang Chen, Chiun-Chieh Yu, Yun-Ru Lai, Pi-Ling Chiang, Wei-Che Lin

**Affiliations:** 1 Department of Diagnostic Radiology, Kaohsiung Chang Gung Memorial Hospital, and Chang Gung University College of Medicine, 123 Ta-Pei Road, Niao-Sung, Kaohsiung, 83305 Taiwan; 2 Department of Neurology, Kaohsiung Chang Gung Memorial Hospital, and Chang Gung University College of Medicine, Kaohsiung, Taiwan; 3 Brain Research Center, National Yang-Ming University, Taipei, Taiwan; 4 Institute of Neuroscience, National Yang-Ming University, Taipei, Taiwan

**Keywords:** Neurodegeneration, Parkinson's disease

## Abstract

Structural covariance assesses similarities in gray matter between brain regions and can be applied to study networks of the brain. In this study, we explored correlations between structural covariance networks (SCNs) and cognitive impairment in Parkinson’s disease patients. 101 PD patients and 58 age- and sex-matched healthy controls were enrolled in the study. For each participant, comprehensive neuropsychological testing using the Wechsler Adult Intelligence Scale-III and Cognitive Ability Screening Instrument were conducted. Structural brain MR images were acquired using a 3.0T whole body GE Signa MRI system. T1 structural images were preprocessed and analyzed using Statistical Parametric Mapping software (SPM12) running on Matlab R2016a for voxel-based morphometric analysis and SCN analysis. PD patients with normal cognition received follow-up neuropsychological testing at 1-year interval. Cognitive impairment in PD is associated with degeneration of the amygdala/hippocampus SCN. PD patients with dementia exhibited increased covariance over the prefrontal cortex compared to PD patients with normal cognition (PDN). PDN patients who had developed cognitive impairment at follow-up exhibited decreased gray matter volume of the amygdala/hippocampus SCN in the initial MRI. Our results support a neural network-based mechanism for cognitive impairment in PD patients. SCN analysis may reveal vulnerable networks that can be used to early predict cognitive decline in PD patients.

## Introduction

Cognitive impairments in Parkinson’s disease (PD) range from mild cognitive impairment (PDMCI) to dementia (PDD)^1^. It is one of the most common and significant non-motor symptoms impacting PD patients’ prognosis, quality of life, while contributing significant costs and burdens to health care systems globally^2^. Although dementia eventually develops in the majority of PD patients, the timing of onset and pace of progression vary greatly among PD patients^3^. Therefore, the ability to identify and predict future cognitive decline is critical for patient management.

Many factors contribute to the progression of cognitive decline in PD patients, such as age, sex, disease duration, and PD motor symptom severity^2^. Recently, molecular biomarkers such as CSF amyloid beta level, and genetic factors have been investigated for their ability to predict cognitive decline in PD patients^3^. In addition to clinical symptoms and molecular biomarkers, neuroimaging provides a noninvasive way to explore the brain of patients with neurodegenerative diseases, both structurally and functionally.

Structural changes of the brain, such as atrophy of the hippocampus, have been shown to be associated with cognitive impairment in PD patients^4^. Furthermore, altered functional connectivity of specific brain regions and networks have been associated with cognitive impairment in PD patients^5,6^. Several studies have indeed explored the potential role of neuroimaging in predicting the progression of cognitive decline in PD patients, with variable results^4,7,8^. According to network-degeneration hypotheses, in neurodegenerative diseases such as PD, disease spread begins at an epicenter most vulnerable to the disease itself and progresses in a network fashion^9,10^. Multiple neural networks are involved in the cognitive impairments observed in PD patients, which are modulated by both dopaminergic and non-dopaminergic systems^11^. Therefore, analyzing the brain on a network basis may provide valuable insights into the development of cognitive impairments in PD patients.

Studies have reported that networks derived from different MRI modalities, such as gray matter (GM) structural covariance and resting-state functional MRI, exhibit similar patterns of disruption in neurodegenerative diseases^10,12^. Structural covariance analyzes similarities in GM between brain regions and can be used to study networks of the brain by assessing differences in covariation among different brain regions across the population. Of note, the biological meaning underlying structural covariance is not completely understood and may be influenced by developmental, genetic, and environmental factors^13^.

The purpose of this study was threefold. First, we aimed to identify the epicenter of PD cognitive impairment and its associated structural covariance network (SCN). Second, we aimed to assess the association between altered SCN and cognitive function domains in PD patients. Third, we aimed to assess the predictability of developing future cognitive impairment in PD patients from altered SCN identified in the aforementioned steps.

## Materials and methods

### Participants

One hundred and one patients (47 males and 54 females; mean age: 61.12 ± 0.84 years) diagnosed with idiopathic PD in accordance with the United Kingdom Brain Bank criteria^14^ and without other neurological disorders or psychiatric diseases were prospectively enrolled in the study at a single tertiary medical center. The Unified Parkinson’s Disease Rating Scale (UPDRS), the modified Hoehn and Yahr Staging Scale, and the Schwab and England Activities of Daily Living Scale were utilized to assess the functional status and disease severity of the patients^15,16^. Fifty-eight healthy control subjects (30 males and 28 females; mean age: 55.17 ± 1.08 years) without neurological disease, psychiatric illness, alcohol or substance abuse, or head injury were recruited as the control group.

### Statement

The hospital’s Institutional Review Committee on Human Research approved the study protocol (Chang Gung Medical Foundation Institutional Review Board; IRB No.: 201601519B0 and 201802352B0C601). Informed consent was obtained from all subjects or their guardians. All methods were carried out in accordance with the relevant guidelines and regulations.

### Neuropsychological testing

A clinical psychologist blinded to each participant’s status performed the Mini-Mental State Examination (MMSE) and a neuropsychological battery of tests using both the Chinese version of the Wechsler Adult Intelligence Scale-III (WAIS-III)^17^ and the Cognitive Ability Screening Instrument (CASI)^18^, with participants undergoing at least 3 tests in each of the following cognitive function domains: attention, executive, speech and language, memory, and visuospatial functions. The tests within cognitive function domains were extracted from subtests of CASI and WAIS-III. In the attention function domain, digit span and letter number sequencing are from WAIS-III while attention and orientation are from CASI. In the executive function domain, digit symbol coding, arithmetic, picture arrangement, and matrix reasoning are from WAIS-III while abstract thinking is from CASI. In the memory function domain, short- and long-term memory are from WAIS-III while information is from CASI. In the speech and language function domain, vocabulary, comprehension, and similarity are from WAIS-III while language and semantic fluency are from CASI. In the visuospatial function domain, picture completion and block design are from WAIS-III while drawing is from CASI.

All PD patients were classified into PDN, PDMCI, or PDD groups using level II criteria in accordance with the Movement Disorder Society Task Force Guidelines. The classification details have been previously described^19^. Among the 101 PD patients, 34 were classified as PDN, 33 were PDMCI, and 34 were PDD. Nineteen PDN patients had another session of neuropsychological testing at the one-year follow-up. The neuropsychological testing items were the same and was performed by the same clinical psychologist.

### Structural MR imaging

#### Image acquisition

The images were acquired using a 3T whole body GE Signa MRI system (GE Healthcare). To diminish motion artifact, each subject’s head was immobilized by foam pillows inside the coil. The T1-weighted structured images were acquired using a 3D-FSPGR sequence. The sequence parameters are as follows: repetition time (TR) = 9.492 ms, echo time (TE) = 3.888 ms, flip angle = 20°, matrix size = 512 × 512, field of view (FOV) = 24 × 24 cm, in-plane spatial resolution: 0.47 × 0.47 mm, and slice thickness: 1.3 mm).

#### Motion assessment

An experienced neuroradiologist visually inspected all anatomical scans to exclude participants with apparent image artifacts or brain abnormalities, including trauma, tumors, hemorrhagic or infarct lesions and motion blur. We also used the MRI Quality Control tool (MRIQC, https://github.com/poldracklab/mriqc)^20^ to check the quality control of the anatomical data. Entropy focus criterion (EFC) value was used as the head motion index in our study.

#### Imaging data pre-processing

The image pre-processing was implemented in Matlab R2016a (Mathworks) via Statistical Parametric Mapping 12 (SPM12; University College London).

During the segmentation process, the T1-weighted structural MR images were segmented in gray matter (GM), white matter (WM), and cerebrospinal fluid (CSF) volumes. The normalization process was based on DARTEL (Diffeomorphic Anatomical Registration Through Exponentiated Lie Algebra) algorithm. MR images from all participants were used to create a study-specific tissue templates which were then transformed to the MNI (Montreal Neurological Institute) space. In the smoothing process, the modulated GM segments were smoothed using an 8-mm full-width-at-half-maximum (FWHM) Gaussian kernel. We set the probability threshold at 0.2 to avoid possible incorporation of tissue with lower GM probability.

### Structural covariance network analysis

**Figure 1:**
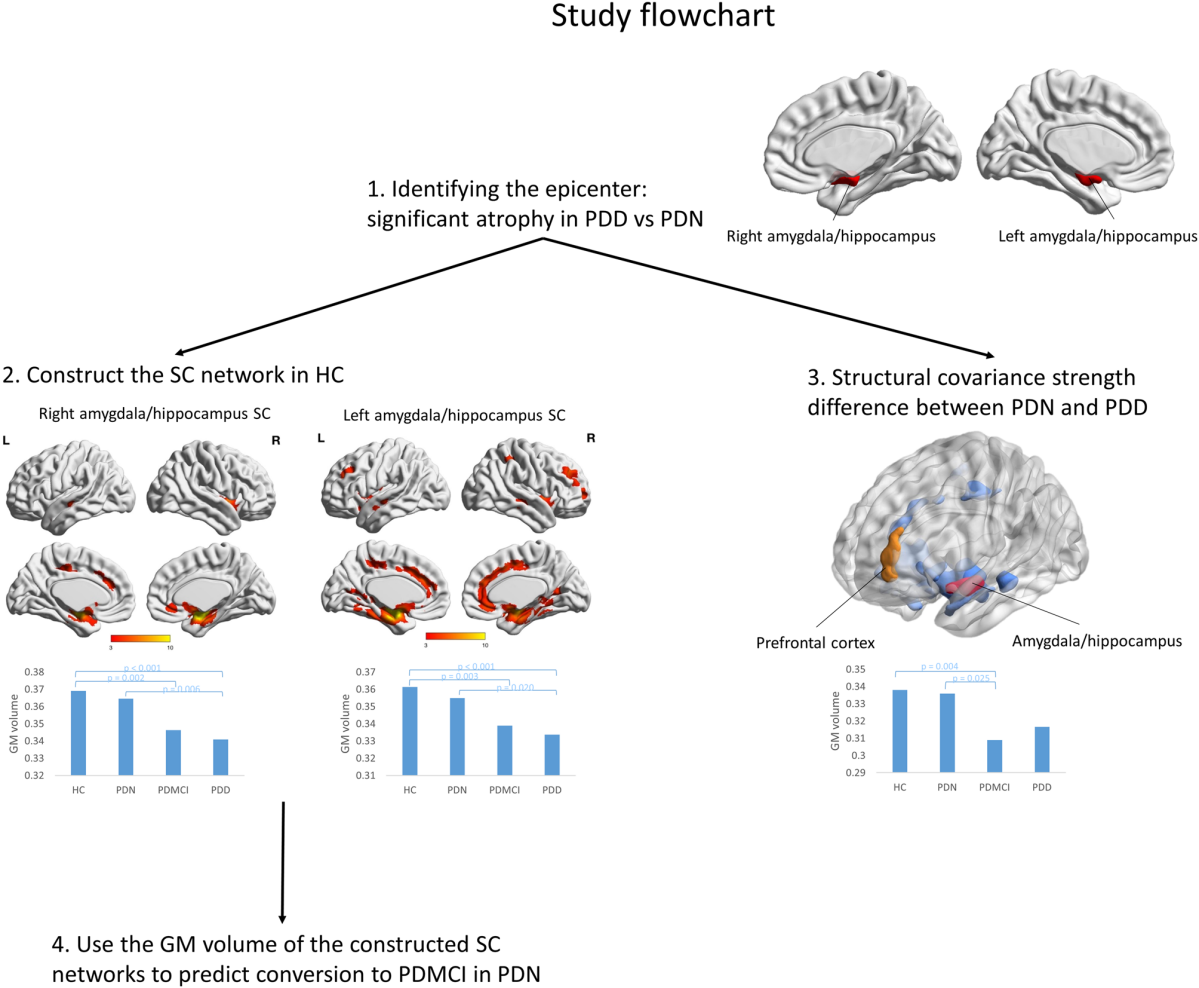
Study flowchart. Step 1. Voxel-wise group comparisons of GMV between PDD and PDN showing atrophy of bilateral amygdala/hippocampus in the PDD group. Step 2. ROIs identified in Step 1 were used to contrast SCN in the normal controls. Step 3. Different structural covariance patterns were analyzed between PDN and PDD (FWE-corrected P < 0.05). Step 4. The GMV of the regions found in Step 2 were calculated and used to predict conversion to PDMCI in PDN patients at 1-year follow-up.

The study flow chart is shown in Fig. 1. In the first step, regions with significantly lower gray matter volume (GMV) between the PDD and PDN groups were chosen as the seeds for investigating the SCNs associated with cognitive status in PD patients. This was done by voxel-wise group comparisons of GMV using full factorial design with age, sex, and total intracranial volume (TIV, calculated as the sum of total voxels of GM, WM, and CSF) as covariates to detect regional GMV differences between the PDD and PDN groups. Results were considered significant under the criteria of family-wise error (FWE)-corrected P value < 0.05 using cluster-extent approach for correcting multiple comparison problem with a cluster size of at least 340 voxels based on the results of the Monte Carlo simulation (the updated 3dFWHMx and 3dClusterSim program implemented in the Analysis of Functional Neuroimages software (AFNI) with the following parameters: single voxel P value < 0.001, FWHM = 8 mm with GM mask, and 10,000 simulations).

In the second step, to construct the SCN in the normal controls, the GMV of the seed ROIs identified in the previous step were calculated in the normal controls, followed by correlation analyses using the extracted GMV as the covariates of interest. This was done using voxel-wise multiple linear regressions performed on the warped GM segments of the normal controls with a regression model including the GMV of the seed ROIs, age, sex, and TIV to account for the confounding effects of overall brain size caused by age, sex, and TIV. A significant positive correlation was deemed as structure covariance.

In the third step, to assess the interaction between cognitive status in PD and structural covariance patterns, regional differences in SC patterns between the PDN and PDD groups were investigated. This was done by a general linear model including a group main effect term, a mean seed ROI volume main effect term, and a group X mean seed ROI volume interaction term for each seed identified in the first step. This design enabled us to investigate regional differences in structural covariance patterns between groups by testing the significance of the interaction term at each voxel.

In the final step, the GMV of ROIs found in the second step were used to predict conversion to PDMCI in the PDN group at follow-up.

### Statistical analysis

#### Analysis of demographic data and longitudinal data

The age data was analyzed using analysis of variance and the sex data was analyzed using Pearson chi-square test. The results were reported as mean ± standard deviation (SD). Disease severity, MMSE, and neuropsychological test scores were analyzed by analysis of covariance (ANCOVA), with subjects’ age, sex, and education level as covariates. Longitudinal data were analyzed using T-test between converters and non-converters. Statistical analysis was performed using the Statistical Product and Service Solutions software version 19 (IBM SPSS). Statistical significance was considered when p value is less than 0.05.

#### Cognitive performance and GMV of ROIs

Because the scoring systems were diverse among tests in each domain of the neuropsychological testing, a weighted domain score was constructed for each patient. Every test scores of the neuropsychological assessment within each domain were normalized and then averaged to make a weighted domain score.

The GMV of ROIs identified in the second and third steps of the Structural covariance network analysis were calculated and partial correlation analysis was done between GMV of ROIs and weighted cognitive domain score with age, sex, UPDRS total score and education level as covariates. The results were considered significant when P value is less than 0.05. Due to the exploratory nature of this study, the results of the correlation analysis were not corrected for multiple comparison.

### Ethical standards and patient consent

We declare that all human and animal studies have been approved by the Institutional Review Board of Chang Gung Memorial Hospital (Chang Gung Medical Foundation Institutional Review Board; IRB No.: 201601519B0 and 201802352B0C601) and have therefore been performed in accordance with the ethical standards laid down in the 1964 Declaration of Helsinki and its later amendments. We declare that all patients gave informed consent prior to inclusion in this study.

## Results

### Baseline clinical characteristics of PD patients and controls

The baseline clinical demographics and neuropsychological assessment scores of all participants are listed in Table 1. The PD patients were significantly older than the controls, with no significant age difference among the PDN, PDMCI, and PDD groups. As expected, PDD patients performed significantly worse than PDN patients in every domain of the neuropsychological testing. Aside from the cognitive performance, PDD patients also had higher UPDRS part III and total scores than PDN patients, indicating that PDD patients had more severe motor symptoms.

**Table 1:** Demographic data and neuro-psychological assessment of PD patients and normal controls.

	Control (n = 58)	PDN (n = 34)	PDMCI (n = 33)	PDD (n = 34)	*P* value
**Clinical demographics**					
Age (year)	55.17 ± 1.08	59.18 ± 1.67	61.36 ± 1.34	62.82 ± 1.30	< 0.001^#$^
Sex (M, F)	30, 28	19, 15	15, 18	13, 21	0.529
Disease duration (year)		2.16 ± 0.28	2.69 ± 0.56	3.25 ± 0.48	0.234
UPDRS I		3.16 ± 0.55	3.18 ± 0.52	4.15 ± 0.52	0.323
UPDRS II		8.64 ± 1.43	10.07 ± 1.37	12.88 ± 1.35	0.104
UPDRS III		20.09 ± 3.00	25.21 ± 2.86	31.05 ± 2.83	0.038^+^
UPDRS total		31.88 ± 4.64	38.45 ± 4.42	48.08 ± 4.37	0.048^+^
Modified H & Y		1.82 ± 0.21	2.28 ± 0.20	2.32 ± 0.20	0.187
S & E		85.25 ± 3.60	80.92 ± 3.43	78.61 ± 3.39	0.421
EFC index	0.68 ± 0.03	0.67 ± 0.03	0.67 ± 0.03	0.67 ± 0.02	0.466
MMSE	28.66 ± 0.40	27.96 ± 0.43	27.07 ± 0.40	21.26 ± 0.44	< 0.001^$^^+^^^^
**Neuro-psychological assessments**					
Attention function					
Digit span	12.41 ± 2.44	11.30 ± 2.44	9.21 ± 2.51	9.61 ± 2.85	< 0.001^#$^^+^
Attention	7.81 ± 0.61	7.74 ± 0.51	7.21 ± 0.99	6.12 ± 1.39	< 0.001^$^^+^^^^
Orientation	17.93 ± 0.32	17.77 ± 1.26	17.30 ± 1.31	15.58 ± 3.60	< 0.001^$^^+^^^^
Letter number sequencing	9.81 ± 4.45	10.63 ± 2.04	8.33 ± 2.77	5.73 ± 2.84	0.006^+^
Executive function					
Digit symbol coding	11.72 ± 2.01	9.77 ± 2.75	7.82 ± 2.71	5.03 ± 2.27	< 0.001*^#$+^^
Arithmetic	11.79 ± 2.21	10.20 ± 2.48	7.76 ± 2.28	6.55 ± 1.80	< 0.001*^#$&+^
Picture arrangement	10.91 ± 3.10	10.37 ± 2.48	7.91 ± 2.61	6.86 ± 2.57	< 0.001^#$&^^+^
Matrix reasoning	11.21 ± 2.87	11.23 ± 2.66	8.64 ± 2.49	6.03 ± 2.44	< 0.001^#$&^^+^^^^
Abstract thinking	10.33 ± 1.28	10.26 ± 1.09	8.55 ± 1.77	6.94 ± 2.24	< 0.001^#$&^^+^^^^
Memory function					
Short-term memory	10.76 ± 1.17	10.55 ± 1.39	9.18 ± 2.52	6.19 ± 2.77	< 0.001^$^^+^^^^
Long-term memory	10.00 ± 0.00	10.00 ± 0.00	9.76 ± 0.83	9.03 ± 1.81	0.020^$^^+^
Information	11.86 ± 2.54	10.27 ± 2.12	8.85 ± 2.48	7.30 ± 1.93	< 0.001*^#$^
Speech and language					
Vocabulary	12.69 ± 2.41	11.65 ± 2.60	8.85 ± 3.27	6.58 ± 2.42	< 0.001^#^^$&^^+^
Comprehension	12.60 ± 2.66	11.33 ± 2.19	8.64 ± 3.23	6.71 ± 2.42	< 0.001^#^^$&^^+^
Language	9.94 ± 0.23	9.92 ± 0.26	9.69 ± 0.97	8.66 ± 1.12	< 0.001^$^^+^^^^
Similarity	11.45 ± 2.23	11.16 ± 2.13	8.45 ± 3.12	6.67 ± 2.64	< 0.001^#^^$&^^+^
Semantic fluency	8.84 ± 1.61	8.39 ± 1.78	7.42 ± 2.15	6.24 ± 2.32	< 0.001^#$^^+^
Visuospatial function					
Picture completion	11.31 ± 2.38	9.94 ± 2.61	8.27 ± 2.81	6.45 ± 2.82	< 0.001^#$^^+^
Block design	11.31 ± 2.76	9.68 ± 2.69	6.88 ± 2.27	5.58 ± 2.53	< 0.001*^#$&+^
Drawing	9.98 ± 0.13	9.58 ± 0.85	9.64 ± 1.27	8.55 ± 1.75	< 0.001^$^^+^^^^

Age data were compared by analysis of covariance (ANCOVA) test. MMSE and neuro-psychological assessment data were compared by analysis of covariance (ANCOVA) after controlling for age, sex, and education.

Sex data were compared by Pearson chi-square test.

The data are presented as mean ± standard error of the mean.

*UPDRS* Unified Parkinson’s disease rating scale, *Modified H & Y* modified Hoehn and Yahr stages, *S & E* Schwab and England activities of daily living scale, *EFC* entropy focus criterion, *MMSE* mini mental state examination.

*p < 0.05 between control and PDN using Bonferroni method.

^#^p < 0.05 between control and PDMCI using Bonferroni method.

^$^p < 0.05 between control and PDD using Bonferroni method.

^&^p < 0.05 between PDN and PDMCI using Bonferroni method.

^+^p < 0.05 between PDN and PDD using Bonferroni method.

^^^p < 0.05 between PDMCI and PDD using Bonferroni method.

### Group comparison of regional GMV between PDD and PDN and construction of SCN

Voxel-wise analysis results of the whole brain with full factorial design are shown in Supplementary Table S1. The PDD patients had significantly lower GMV in the bilateral amygdala/hippocampus compared to PDN patients. These two regions were used as seeds separately to construct SCNs in the healthy controls. The constructed SCNs from both seeds were similar and involved bilateral frontal, temporal, and cingulate cortex as shown in Fig. 1 and Supplementary Table S2A,B.

### Connectivity interactions between PDD and PDN with the epicenter

The interaction of cognitive status with regard to covariance strength difference is shown in Table 2. Within the SCN anchored to the right amygdala/hippocampus seed, significantly increased structural covariance was observed in the PDD group compared to the PDN group. The peak cluster showing interaction lies in the left prefrontal cortex. There was no significant decreased structural covariance in the PDD group compared to the PDN group. Within the SCN anchored to the left amygdala/hippocampus seed, there was no significant interaction.

**Table 2:** Connectivity interactions between PDD and PDN with epicenters.

Epicenter	Cluster size	Anatomical region	MNI coordinates			Maximum T values
			x	y	z	Within clusters
**PDD > PDN**						
R amygdala/hippocampus	500	L anterior prefrontal cortex	− 30	56	12	4.65
		L dorsolateral prefrontal cortex	− 32	50	26	
		L dorsolateral prefrontal cortex	− 38	54	18	
L amygdala/hippocampus	NA					
**PDD < PDN**						
R amygdala/hippocampus	NA					
L amygdala/hippocampus	NA					

*GMV* gray matter volume, *R* right, *L* left.

### Correlation between cognitive function and volumes of SCNs and prefrontal cortex

**Figure 2:**
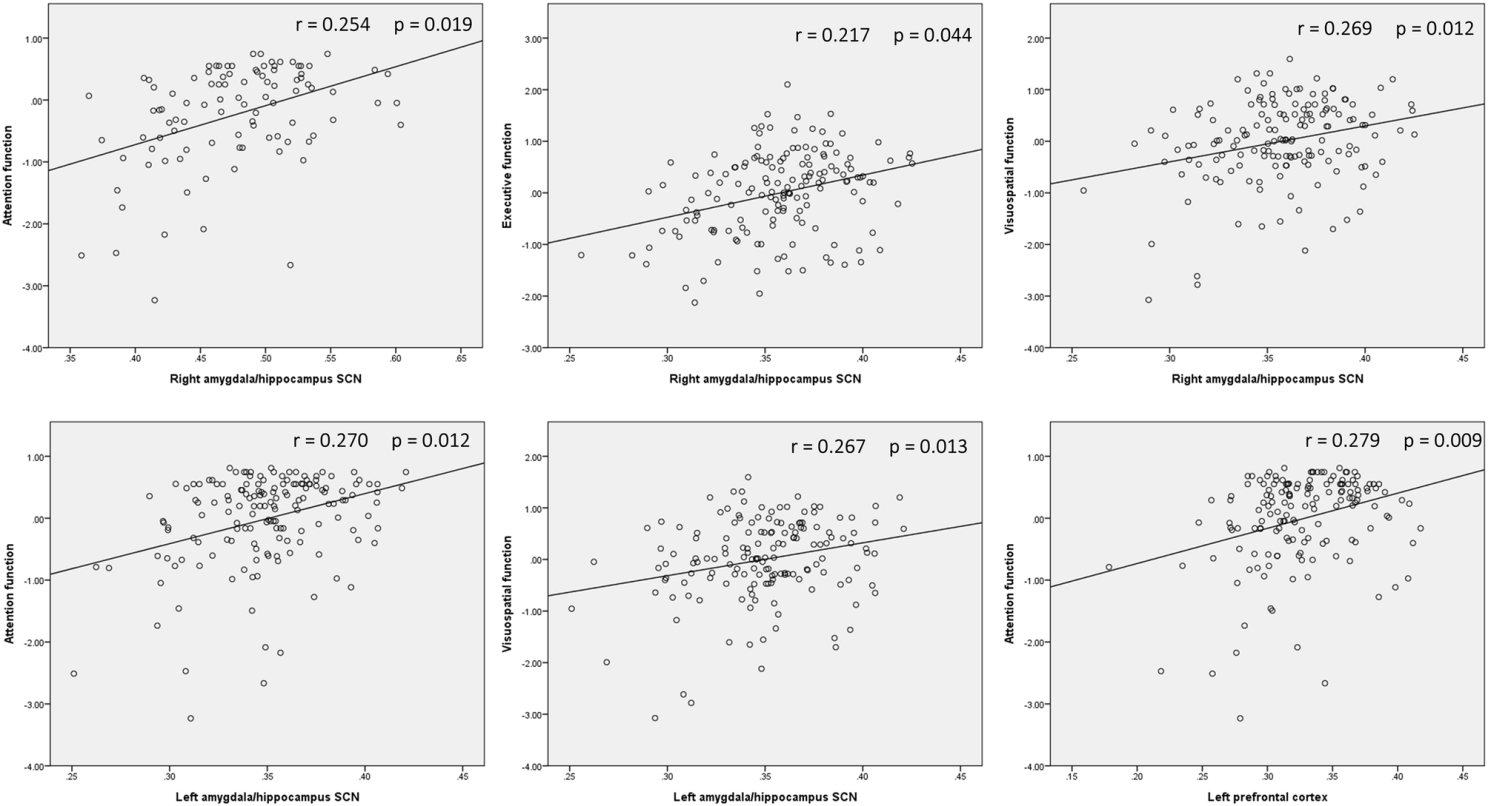
Correlation between cognitive performance and ROIs. The GMV of the SCN constructed from the right amygdala/hippocampus is correlated with with attention, executive, and visuospatial domains. The GMV of the SCN constructed from the left amygdala/hippocampus is correlated with attention and visuospatial domains. The GMV of the left prefrontal cortex is correlated with attention domain.

The partial correlation analysis between the different cognitive domains and volumes of SCNs and prefrontal cortex is shown in Fig. 2. The GMV of the SCN constructed from the right amygdala/hippocampus seed is associated with attention, executive, and visuospatial function. The GMV of the SCN constructed from the left amygdala/hippocampus seed is associated with attention, and visuospatial function. The GMV of the prefrontal cortex is associated with attention function.

### Longitudinal results on the GMV of SCNs and left prefrontal cortex

**Figure 3:**
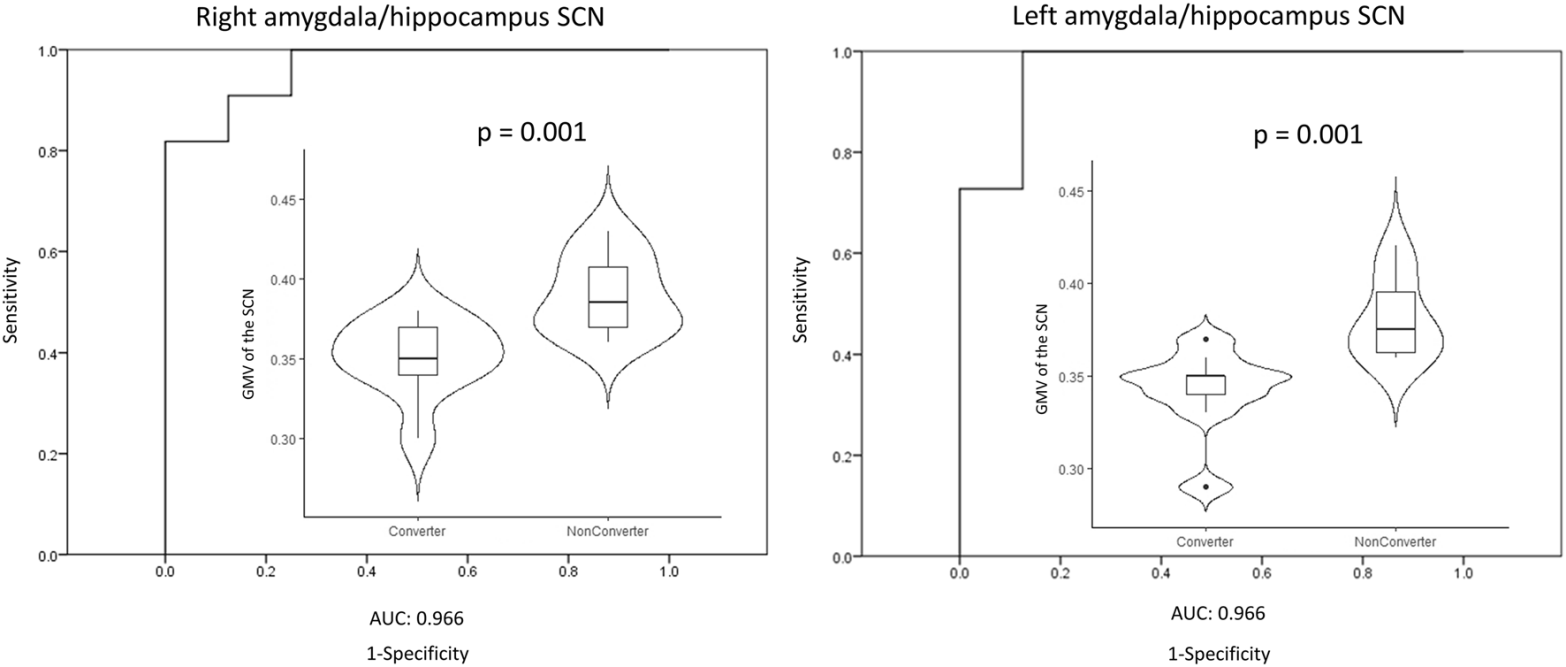
Longitudinal results on the GMV of bilateral amygdala/hippocampus SCN. ROC curve with AUC = 0.966 and 0.966 respectively for GMV of right and left amygdala/hippocampus SCN of discriminating between PD converter and PD non-converter. Violin plots show the GMV for each ROI in PD converter and PD non-converter.

We further evaluated if the GMV of the identified SCNs could potentially predict the progression to cognitive decline in PDN patients. Of the 19 PDN patients that received a follow-up visit 1 year after the initial assessment, nine patients progressed to PDMCI (PD converter) while the other 10 patients remained in normal cognition status (PD non-converter). As shown in Fig. 3, in the initial MRI, there were already significant differences in the GMV of the SCNs constructed from the bilateral amygdala/hippocampus seed.

## Discussion

The aims of this study were to identify cognitive impairments associated with SCN alterations in PD patients, and to assess the ability of SCN alterations to predict future cognitive decline in PD patients with normal cognition. As such, we identified hippocampus/amygdala atrophy in PDD patients. Since this region is the most atrophied region for cognitive impairment in PD patients, we used it to construct the cognitive impairment-associated SCN, which showed progressive atrophy with disease progression in PD patients. Longitudinally, alteration in the cognitive impairment-associated SCN can predict future cognitive decline in PD patients with normal cognition. To further comfirm the effect of prediction, a re-analysis over step 1 and 2 excluding the 19 PDN patients that received follow-up showed similar atrophied region and associated SCNs with similar prediction ability (results not shown).

As compared to the PDN group, our results showed that the PDD group had significant atrophy of the bilateral hippocampus and amygdala, which are frequently reported as atrophied regions in both PDD and PDMCI patients^21–24^. Notably, a study by Melzer et al. showed that progression of PD to PDMCI to PDD correlates with increasing atrophy of the amygdala and hippocampus^21^. Therefore, these regions and the integrity of their associated networks may be important for maintaining intact cognitive function in PD patients. To assess PD cognitive impairment-related network degeneration, we used the amygdala and hippocampus regions as the seed to construct the SCN in healthy controls. The resultant network involves structures of the medial temporal lobe, cingulate gyrus, prefrontal cortex, and part of the parietal lobe. The findings are similar to those of a previous SCN study^25^ and the functional network using resting-state fMRI conducting on normal population^26^. The amygdala/hippocampus network identified in this study overlaps that of the limbic network, which plays major roles in emotion, cognition, and behavior^27^.

Although SCNs of the brain mimic those of function networks, the biological meaning of SCNs remain unclear. The observed covariance between GM structures are likely attributed to the combined effect of synaptic connectivity between the brain regions, coordinated neurodevelopment, and genetic factors^13^. In neurodegenerative diseases, the damaged areas are often regions that are highly structurally correlated in healthy individuals^10^.

A recent study comparing PD patients and healthy controls demonstrated SCN atrophy in PD patients^28^. Furthermore, the study showed that the degree of atrophy in each brain region correlated with its functional and anatomical proximity to the substantia nigra, which supports the role of trans-neuronal spread and the network-degeneration mechanism in PD patients^28^. In the present study, by measuring GM volume of the amygdala/hippocampus network across different groups, we identified progressive atrophy of the network associated with cognitive status deterioration. This result provides further support of the network-degeneration mechanism in the cognitive impairment of PD patients.

Using seed-based covariance analysis, we found increased covariance between the amygdala/hippocampus and prefrontal cortex in PDD patients compared to PDN patients. The amygdala connects to the prefrontal cortex through the uncinate fasciculus as part of the temporo-amygdala-orbitofrontal network. The increased covariance between these two regions may be explained by correlated GM loss targeted by the same degenerative process. The amygdala and prefrontal cortex are often affected in neurodegenerative dementia, such as that observed in Alzheimer's disease patients^29,30^, while the involvement of the amygdala-orbitofrontal network is associated with semantic deficits^27^. The prefrontal cortex receives neural connections from the nigrostriatal dopamine network, mesocortical dopamine network, and noaradrenergic network. In PDD patients, damage to the neural pathway of these networks leads to dysfunction of the prefrontal cortex, which results in executive dysfunction and attention deficits^11^. This may explain the significant correlation between prefrontal region atrophy and attention function impairments identified in the present study.

Neuropathological studies have demonstrated that the amount of Lewy-related pathology deposition in the neocortex and limbic system is the primary predictor for development of dementia in PD patients^11^. Of note, higher densities of Lewy-related pathology and amyloid-β senile plaques are found in the hippocampus of PDD patients compared to PDN patients^31,32^, which may cause the medial temporal lobe structure atrophy often found in PDD patients^11^. In the present study, by measuring the GMV of the amygdala/hippocampus SCN in PDN patients, we effectively identified patients at risk of developing cognitive impairment in the future. However, larger trials are required to further confirm the ability of the amygdala/hippocampus network GMV to predict development of cognitive impairment in PDN patients.

### Limitations

There are indeed several limitations to this study. Firstly, SCN cannot replace the functional network, thus future studies comparing changes in the functional network and SCNs in PD patients with cognitive impairments are necessary. Secondly, although all participants in this study underwent comprehensive neuropsychological testing, the memory domain tests may lack ample thoroughness, thereby resulting in less discriminability among the different groups. This could be a reason for a non-significant correlation between memory function and SCN GM density in PD patients. Thirdly, the PD patients are significantly older than controls while PD subgroups showed no significant age difference. However, since the main comparison are among different subgroups of PD and all analyses were controlled for age, we therefore belief that the main results in this study are not significantly affected by this factor. Lastly, the number of patients included in each group and in the longitudinal follow-up was relatively small. Future study with larger dataset can perform separate analysis for finding the most atrophied region and testing the region for interaction. Future large-scale longitudinal studies with possible integration of clinical and laboratory biomarkers may provide a better predictability model for application in clinical practice.

In conclusion, our results provide further evidence of a neural network-based mechanism in the development of cognitive impairment in PD patients. Structural covariance network analysis may effectively identify vulnerable networks which can be used to predict cognitive decline in PD patients.

## Supplementary Information

Supplementary Tables.

## Data Availability

The data that support the findings of this study are available on request from the corresponding author. The data are not publicly available due to privacy or ethical restrictions.
