# Supplementary material for: The corticolimbic structural covariance network as an early predictive biosignature for cognitive impairment in Parkinson's disease
Source: Sci Rep. 2021 Jan 13;11:862. doi: 10.1038/s41598-020-79403-x (PMC7806769; doi:10.1038/s41598-020-79403-x)
Supplement: Supplementary file 1 — Supplementary Tables. [file 41598_2020_79403_MOESM1_ESM.docx]

**The Corticolimbic Structural Covariance Network as an Early Predictive Biosignature for Cognitive Impairment in Parkinson's Disease**

Yueh-Sheng Chen*^1^*; Hsiu-Ling Chen *^1^*; Cheng-Hsien Lu*^2^;* Chih-Ying Lee*^1^*; Kun-Hsien Chou*^3,4^*; Meng-Hsiang Chen*^1^*; Chiun-Chieh Yu*^1^*; Yun-Ru Lai*^2^*; Pi-Ling Chiang*^1^*; Wei-Che Lin*^1^***^🖂^**

*^1^ Department of Diagnostic Radiology, Kaohsiung Chang Gung Memorial Hospital, and Chang Gung University College of Medicine, Kaohsiung, Taiwan*

*^2^ Department of Neurology, Kaohsiung Chang Gung Memorial Hospital, and Chang Gung University College of Medicine, Kaohsiung, Taiwan*

*^3^ Brain Research Center, National Yang-Ming University, Taipei, Taiwan.*

*^4^ Institute of Neuroscience, National Yang-Ming University, Taipei, Taiwan*

^🖂^Wei-Che Lin, MD, PhD, Department of Diagnostic Radiology, Kaohsiung Chang Gung Memorial Hospital, Chang Gung University College of Medicine, 123 Ta-Pei Road, Niao-Sung, Kaohsiung 83305, Taiwan; Tel: +886-7-731-7123 ext 3027; Fax: +886-7-731-7123 ext 2523; E-mail: linwc137@gmail.com

**Supplementary Table 1. Anatomical regions showing reduced GMV in PDD vs PDN**

|  |  | MNI coordinates | | | Maximum T values |
| --- | --- | --- | --- | --- | --- |
| Cluster size | Anatomical region | x | y | z | within clusters |
| 997 | R amygdala | 34 | 4 | -15 | 4.67 |
|  | R hippocampus | 21 | -10 | -14 |  |
| 534 | L amygdala | -18 | 2 | -18 | 4.07 |
|  | L hippocampus | -32 | -12 | -15 |  |

Abbreviations: GMV, gray matter volume; R, right; L, left

**Supplementary Table 2A. Anatomical regions of normal controls that covaried with right amygdala/hippocampus**

|  |  | MNI coordinates | | | Maximum T values |
| --- | --- | --- | --- | --- | --- |
| Cluster size | Anatomical region | x | y | z | within clusters |
| 12122 | R amygdala | 26 | -3 | -15 | 23.68 |
|  | R hippocampus | 28 | -6 | -15 |  |
|  | R putamen | 36 | 3 | -9 |  |
| 364 | L middle temporal gyrus | -58 | -21 | -12 | 5.24 |
|  | L superior temporal gyrus | -54 | -18 | -4 |  |
| 461 | L midcingulate area | -12 | -22 | 48 | 4.47 |
|  | L midcingulate area | -10 | -32 | 39 |  |
|  | L midcingulate area | -10 | -12 | 39 |  |
| 613 | L anterior cingulate gyrus | -10 | 34 | 26 | 4.29 |
|  | L midcingulate area | -4 | 21 | 38 |  |
|  | L midcingulate area | -8 | 2 | 40 |  |

Abbreviations: R, right; L, left

**Supplementary Table 2B. Anatomical regions of normal controls that covaried with left amygdala/hippocampus**

|  |  | MNI coordinates | | | Maximum T values |
| --- | --- | --- | --- | --- | --- |
| Cluster size | Anatomical region | x | y | z | within clusters |
| 40539 | L hippocampus | -24 | -9 | -16 | 36.08 |
|  | R hippocampus | 20 | -9 | -12 |  |
|  | R parahippocamal gyrus | 21 | 4 | -20 |  |
| 1607 | R middle frontal gyrus | 30 | 38 | 34 | 5.68 |
|  | R middle frontal gyrus | 27 | 52 | 0 |  |
|  | R middle frontal gyrus | 33 | 44 | 18 |  |
| 391 | R inferior parietal lobule | 36 | -40 | 51 | 4.90 |
|  | R inferior parietal lobule | 58 | -46 | 45 |  |
|  | R inferior parietal lobule | 45 | -48 | 51 |  |
| 500 | L middle frontal gyrus | -28 | 38 | 28 | 4.76 |
|  | L middle frontal gyrus | -39 | 30 | 36 |  |
| 387 | L middle temporal gyrus | -60 | -16 | -8 | 4.70 |
|  | L middle temporal gyrus | -54 | -12 | -20 |  |

Abbreviations: R, right; L, left
